# Comprehensive lnc-RNAs expression profiles in uremic cardiomyopathy before and after renal transplantation

**DOI:** 10.3389/fcvm.2026.1718529

**Published:** 2026-03-12

**Authors:** Xiaoxia Song, Sijia Zhao, Pin Sun, Xiaofei Chen, Hongyang Wang, Yuanyuan Meng, Mingming Lin, Xiaofan Wang, Tao Yu, Zhirong Jiang

**Affiliations:** 1 Department of Cardiac Ultrasound, The Affiliated Hospital of Qingdao University, Qingdao, Shandong, China; 2 Department of Kidney Transplantation, The Affiliated Hospital of Qingdao University, Qingdao, Shandong, China; 3 Institute for Translational Medicine, The Affiliated Hospital of Qingdao University, Qingdao, China

**Keywords:** chronic kidney disease, echocardiography, lncRNAs, renal transplantation, uremic cardiomyopathy

## Abstract

**Background:**

Chronic kidney disease (CKD) significantly contributes to increased cardiovascular morbidity and mortality. CKD-induced cardiac remodeling, clinically termed uremic cardiomyopathy (UCM), manifests as morphological and physiological alterations in the myocardium. While renal transplantation is known to mitigate uremia's effects on myocardial remodeling, improve cardiac function, and reverse damage, its underlying biological mechanism remains unclear. Exosomes mediate inter-organ communication, with their nucleic acid components serving as key regulatory molecules. Emerging evidence indicates long non-coding RNAs (lncRNAs) critically participate in cardiac disease mechanisms.

**Objective:**

This study aimed to analyze the expression profile of blood exosome-derived lncRNAs in UCM patients before and after transplantation to explore lncRNA expression patterns, regulatory mechanisms, and identify key lncRNAs involved in UCM pathogenesis.

**Patients and methods:**

Our study utilized high-throughput RNA sequencing to identify differentially expressed long non-coding RNAs (lncRNAs) in patients with uremic cardiomyopathy (UCM) before and after kidney transplantation. We analyzed the differential expression of lncRNAs from multiple perspectives, including expression profiles, lncRNA-mRNA interaction networks, and enriched GO and KEGG pathways, followed by validation through RT-qPCR.

**Results:**

Gene sequencing revealed 769 dysregulated lncRNAs [440 downregulated, 329 upregulated; log2(fold change) > 2.0, *p* < 0.05]. Computational biological analysis implicated p53 and FoxO signaling pathways in UCM pathogenesis. Differential lncRNA-mRNA interaction networks identified three potential UCM-associated genes: lnc-LOC105379080, lnc-LOC101927608, and lnc-LOC105369947. RT-qPCR validation confirmed significant upregulation of lnc-LINC02194 (*p* = 0.0003), and lnc-MYOSLID-AS1 (*p* = 0.0030), and significant downregulation of lnc-LINC01229 (*p* = 0.0052).

**Conclusion:**

LncRNAs show significant correlation with UCM progression before and after renal transplantation. Lnc-LINC02194 may represent a candidate therapeutic target for UCM.

## Introduction

The risk of mortality due to cardiovascular disease among patients with uremia is approximately six times higher than in patients solely experiencing end-stage renal disease (1). Secondary hemodynamic disturbances related to uremic cardiomyopathy (UCM) represent a primary cause of mortality. Cardiac hypertrophy, diastolic or systolic dysfunction, and myocardial fibrosis in uremia patients may induce severe cardiac stress and injury. These pathological changes lead to complications such as coronary atherosclerosis and ischemic heart injury (2–4), which are frequently observed in patients with established kidney failure. At present, dialysis and renal transplantation constitute the principal therapeutic approaches for uremic patients. Current researches indicate that renal transplantation mitigates cardiac injury, reduces cardiac hypertrophy, improves diastolic function, and substantially enhances overall cardiac performance (5, 6). However, severe cardiac dysfunction remains a significant contraindication for renal transplantation (5). Thus, elucidating the underlying mechanisms by which renal transplantation improves cardiac function is imperative, facilitating timely interventions in cardiac dysfunction among uremic patients, and enhancing our understanding of heart-kidney interrelations.

Exosomes are small lipid vesicles of 40–100 nm secreted by cells, widely distributed in organisms, and play crucial roles in signal transduction and intercellular communication. Exosomes derived from mesenchymal stem cells (MSCs) have been shown to enhance cell proliferation and angiogenesis efficacy, thereby enhancing myocardial repair (7). In the kidney, exosomes can be released by cells as podocytes, pass through the renal ducts, and be absorbed by receptor epidermal cells in the collecting ducts, or affect them by secreting their contents (8, 9).

During the biogenesis of exosomes, they incorporate nucleic acids, lipids, and soluble proteins. Among these cargoes, nucleic acids exert transcriptional regulation on recipient cells, thereby modulating cellular functions and mediating biological activities (8, 10). Long-chain non-coding RNA (lncRNA) constitutes a category of non-coding RNA molecule, typically >200 nucleotides long. Although it does not directly encode proteins, it exerts critical involvement in the pathogenesis of diverse diseases through modulation of gene expression (11, 12). Extracellular vesicle lncRNA has the potential as a diagnostic and prognostic biomarker (13). For example, extracellular vesicles carrying LncRNA TUG1 play a regulatory role in anti angiogenesis and remote ischemic preconditioning in myocardial infarction, are potential therapeutic targets for percutaneous coronary intervention after myocardial infarction (with or without reperfusion) (14), and can reduce renal ischemia/reperfusion injury by interacting with SRSF1 to modulate ASCL4-mediated iron-toxic and lipotoxic toxicity (15). Overexpression of exosomal LncRNA KLF3-AS1 in heart failure patients can reduce Ang II induced myocardial hypertrophy and is a potential diagnostic biomarker for heart failure (16). Differential expression of lncRNAs can affect cardiomyocyte apoptosis, mitochondrial division, and autophagy both *in vitro* and *in vivo* (17). Differential expression of lncRNA CASC2 and ENST00000453774.1 affect the development of renal interstitial inflammation and fibrosis (18, 19). LncRNAs are also implicated in the development and progression of cardiovascular disorders. It is reported that lncRNA is upregulated in serum and coronary plaque of atherosclerosis patients (20–24). LncRNA overexpression induces plaque formation, while low expression reduces plaque accumulation (25). LncRNA ANRIL promotes plaque formation and contributes to atherosclerosis by regulating the expression of inflammatory factors (26). LncRNA CHRF aggravates myocardial cell apoptosis by adsorbing miR-489, contributing to myocardial infarction (27). LncRNAs are key regulators in various biological pathways. However, the role of renal failure in UCM and the differential expression of lncRNAs after renal transplantation remain understudied.

We conducted high-throughput sequencing on blood derived exosomes to identify differentially expressed lncRNAs (DElnRNAs) and performed Gene Ontology (GO), Kyoto Encyclopedia of Genes and Genomes (KEGG) enrichment and Gene Set Enrichment Analysis (GSEA) on these lncRNAs and their target genes, providing detailed insights into their functions and regulatory pathways. We identified several DElncRNAs potentially associated with the improvement of cardiac function after renal transplantation. These findings provide insights into the potential molecular mechanisms underlying cardiac improvement following renal transplantation.

## Materials & methods

### Sample collection

From May 2021 to May 2022, peripheral venous samples were obtained from UCM patients pre-and post-renal transplantation at the Qingdao University Affiliated Hospital. UCM patients are defined as individuals suffering from CKD accompanied by left ventricular hypertrophy (LVH) and decreased diastolic function, with or without systolic dysfunction (3, 28). The screening criteria included uremic patients presenting with cardiac diastolic dysfunction and/or systolic dysfunction in the absence of other documented cardiac diseases. Systolic dysfunction was diagnosed in UCM patients when absolute value of left ventricular global longitudinal strain (LVGLS) measured <18% or left ventricular ejection fraction (LVEF) fell below 55%. Decreased diastolic function in UCM was diagnosed when at least 3 echocardiographic abnormalities were present: peak early mitral inflow velocity to early diastolic mitral annular e’ velocity ratio (E/e’) >15; reduced septal e’ velocity (<7 cm/s) or lateral e’ velocity (<10 cm/s); tricuspid regurgitation peak velocity >2.8 m/s; increased left atrial volume index (>34 mL/m^2^), and isovolemic relaxation time (IVRT) >90 ms. Patients with other undocumented cardiac disorders and abnormal thyroid functions were excluded from this study. The study complied with the Declaration of Helsinki. The study was approved by the Ethics Committee of the Affiliated Hospital of Qingdao University (No. QYFY WZLL 29892). Written informed consents were obtained from all participants following full study disclosure prior to inclusion.

### Exosome isolation and purification

Serum-derived exosomes were isolated from human serum using differential ultracentrifugation. Briefly, the serum was thawed at 37 °C and centrifuged at 2,000 × g for 30 min at 4 °C to remove cells and debris. The supernatant was then centrifuged at 10,000 × g for 45 min at 4 °C to pellet larger vesicles. The resulting supernatant was filtered through a 0.45 μm membrane and subjected to ultracentrifugation at 100,000 × g for 90 min at 4 °C (Hitachi CP100MX) to pellet exosomes. The pellet was washed once with 10 mL of cold 1× PBS and centrifuged again under the same conditions. Finally, the purified exosome pellet was resuspended in 200 μL of cold 1× PBS and stored at −80 °C until further analysis. The isolated exosomes were characterized by transmission electron microscopy (TEM, Hitachi HT-7700), nanoparticle concentration and size distribution analysis (NanoFCM N30E), and detection of exosomal protein markers (CD9 and TSG101) by western blot. Protein concentration was determined using a BCA assay (Beyotime).

### Echocardiography

Initially, routine and three-dimensional echocardiographic images of UCM patients were acquired pre- and post-renal transplantation using a Philips EPIQ 7C color Doppler ultrasound system equipped with S5-1 and X5-1 probes. Subsequently, the offline datasets were processed on a TomTec workstation to generate three-dimensional speckle-tracking analyses for these patients.

### High-throughput sequencing

We collected a total of 78 UCM patients and randomly selected 4 to collect peripheral venous samples both pre- and post-renal transplantation for next-generation sequencing: Group B (pre-transplant, *n* = 4) and Group A (post-transplant, *n* = 4). We performed whole transcriptome sequencing and comprehensive analysis on all venous blood samples at Ribobio Co. Ltd (Guangzhou, China).

### Construction of lncRNA sequencing libraries

Total RNA was isolated utilizing Magzol Reagent (Magen, China). RNA quantity and quality were assessed with the Qubit (Thermo Fisher Scientific, USA) and the Agilent 2200 TapeStation (Agilent Technologies, USA). Thereafter, first- and second-strand complementary DNA (cDNA) synthesis was performed, and adapter ligation plus limited-cycle PCR enrichment were conducted in accordance with the NEBNext® UltraTM RNA Library Prep Kit for Illumina protocol (NEB, USA). Final library size distribution and concentration were again assessed on the Agilent 2200 TapeStation and Qubit.

### Differential expression analysis

DESeq2 was employed for differential expression analysis based on read counts, with Benjamini–Hochberg multiple test correction method. Genes exhibiting an absolute fold change >2 and an adjusted *p*-value < 0.05 were identified as significantly differentially expressed.

### GO and KEGG

The “clusterProfiler” package in R Bioconductor was employed to identify and visualize enriched GO terms and KEGG pathways among all DElnRNAs. For functional annotation, DElnRNAs with |log2(Fold Change)| > 1 and adjusted *P*-value < 0.05 were subjected to enrichment analysis. GO functional categorization was performed using the hypergeometric distribution method, with the entire genome of the species serving as the background reference. Significant terms were identified at a threshold of *P* < 0.05. KEGG pathway analysis was conducted to elucidate signaling transduction and disease-related mechanisms. Statistical significance was determined using the Fisher Exact Test (*P* < 0.05). The distribution and significance of DElnRNAs across KEGG categories were thereby characterized.

### GSEA

We conducted GSEA to ensure that genes with substantial biological significance, but not dramatically differentially expressed, are not overlooked, and identify the key pathways involved in the development and changes of uremic cardiomyopathy before and after renal transplantation.

### Connectivity networks mapping relationships among DElncRNAs

A lncRNA-mRNA co-expression network was built to investigate DElncRNA functions and mRNA interactions. Transcript associations were evaluated through Pearson correlation analysis, with stringent filtering criteria applied (correlation coefficient > 0.85; adjusted *p*-value < 0.05) to establish significant co-expression relationships.

### RT-qPCR

The lncRNAs with most remarkable differential expression and high expression from the RNA sequencing results were selected for RT-qPCR detection. We extracted total RNA from venous blood samples of 8 UCM patients pre- and post-renal transplantation (*n* = 8) using TRIZOL reagent (Vazyme, China). Subsequently, cDNA synthesis was performed via reverse transcription. RT-qPCR was conducted using SYBR Green qPCR Mix (Yeasen, China) on Roche LightCycler 96 system (Roche Technologies, Inc.). GAPDH was considered as an endogenous control gene to normalize lncRNA expression data. Quantification via the 2-ΔΔCT comparative threshold cycle method was performed to analyze the results. Data were derived from the average of two independent experiments.

### Statistical analysis

Independent experiments were performed at least three times. Statistical analyses were conducted using SPSS (version 22.0, IBM, Armonk, NY, USA) and R (R Core Team, 2024, version 4.4.1). For differential expression analysis of lncRNAs, a paired design was implemented to account for individual patient variability, with patient ID serving as a blocking factor. Identification of differentially expressed lncRNAs (DElncRNAs) was achieved through RNA sequencing analysis following the approach described by Anders and Huber (29), utilizing the DESeq2 package (version 1.18.1). To control the false discovery rate (FDR) associated with the limited sample size (*n* = 4 pairs), multiple testing correction was performed using the Benjamini–Hochberg (BH) procedure. Statistical significance was defined as adjusted *P*-value < 0.05 with a |log2(fold change)| > 2.0. For the RT-qPCR validation, data were expressed as means ± standard deviation (SD). To ensure robustness, we performed normality tests (Shapiro–Wilk test) on the residuals before applying parametric tests. Comparisons between pre- and post-treatment groups were conducted using the paired Student's t-test when data were normally distributed; otherwise, the Wilcoxon signed-rank test was used. To account for multiple comparisons across the validation panel, we applied the Benjamini–Hochberg (BH) procedure to adjust *P*-values, defining statistical significance at an adjusted *p* < 0.05. Technical replicates were addressed by computing the mean of biological replicates to minimize variability. The detailed clinical information of each sequencing patient can be found in Supplementary Table S1.

## Results

### Echocardiography

**Figure 1:**
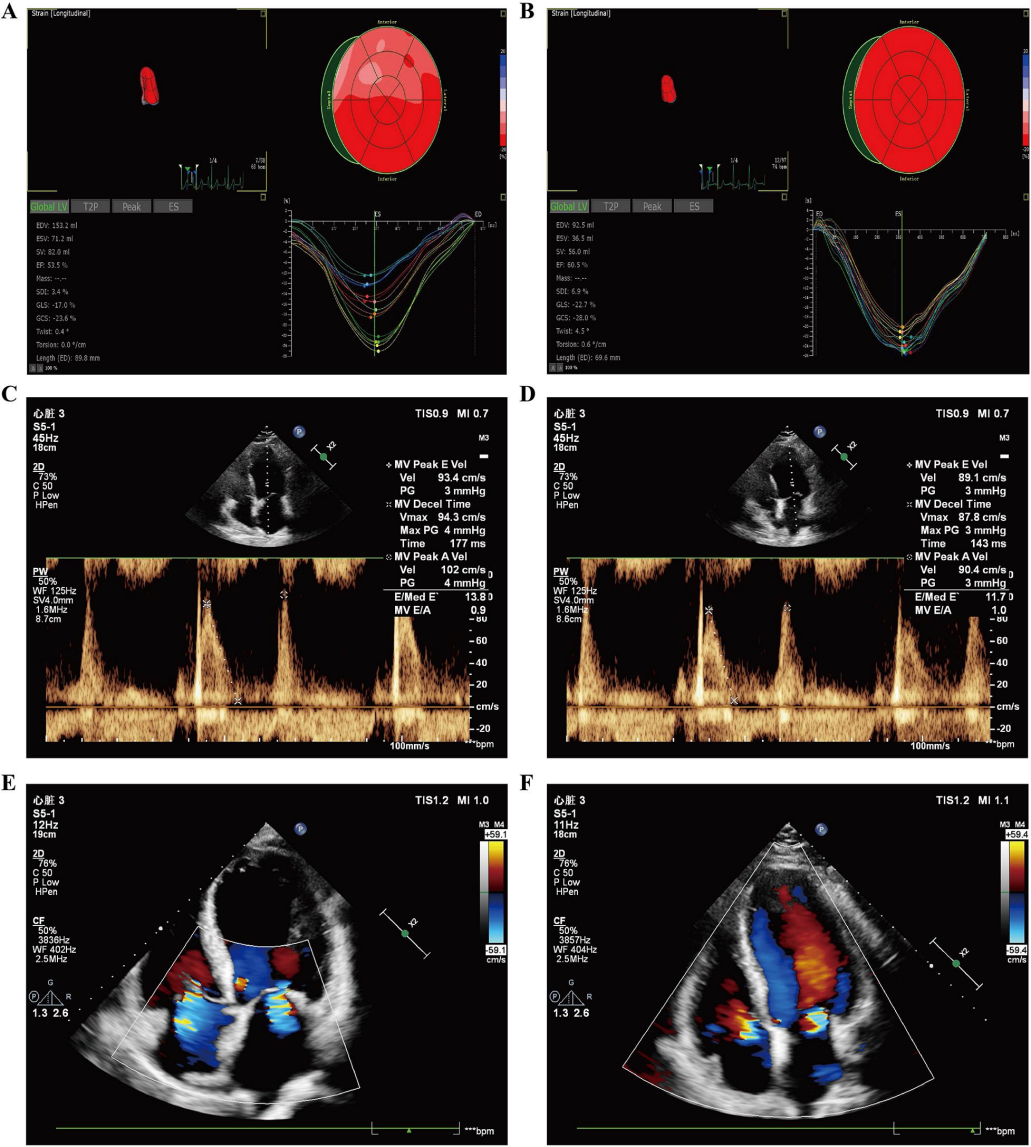
Left ventricular diastolic function and systolic function measured by three-dimensional speckle tracking echocardiography in UCM patients before and after renal transplantation. **(A)** Left ventricular three-dimensional volume, ejection fraction, strain parameters, and strain curves before renal transplantation. **(B)** Left ventricular three-dimensional volume, ejection fraction, strain parameters, and strain curves of the same patient after renal transplantation. **(C)** Left ventricular diastolic function before renal transplantation. **(D)** Improved left ventricular diastolic function of the same patient after renal transplantation. **(E)** Mitral regurgitation volume before renal transplantation. **(F)** Reduced mitral regurgitation volume of the same patient after renal transplantation.

Results displayed that both contractile properties and diastolic compliance of left ventricular were improved in UCM patients after renal transplantation. Greater magnitude (more negative) of left ventricular global longitudinal strain reflected improved systolic performance (Figures 1A,B), while decreases in E/e’ ratios and increases in E/A ratios both indicated enhanced diastolic function (Figures 1C,D). Mitral and tricuspid regurgitation decreased following renal transplantation (Figures 1E,F).

### Isolation and characterization of serum exosomes

**Figure 2:**
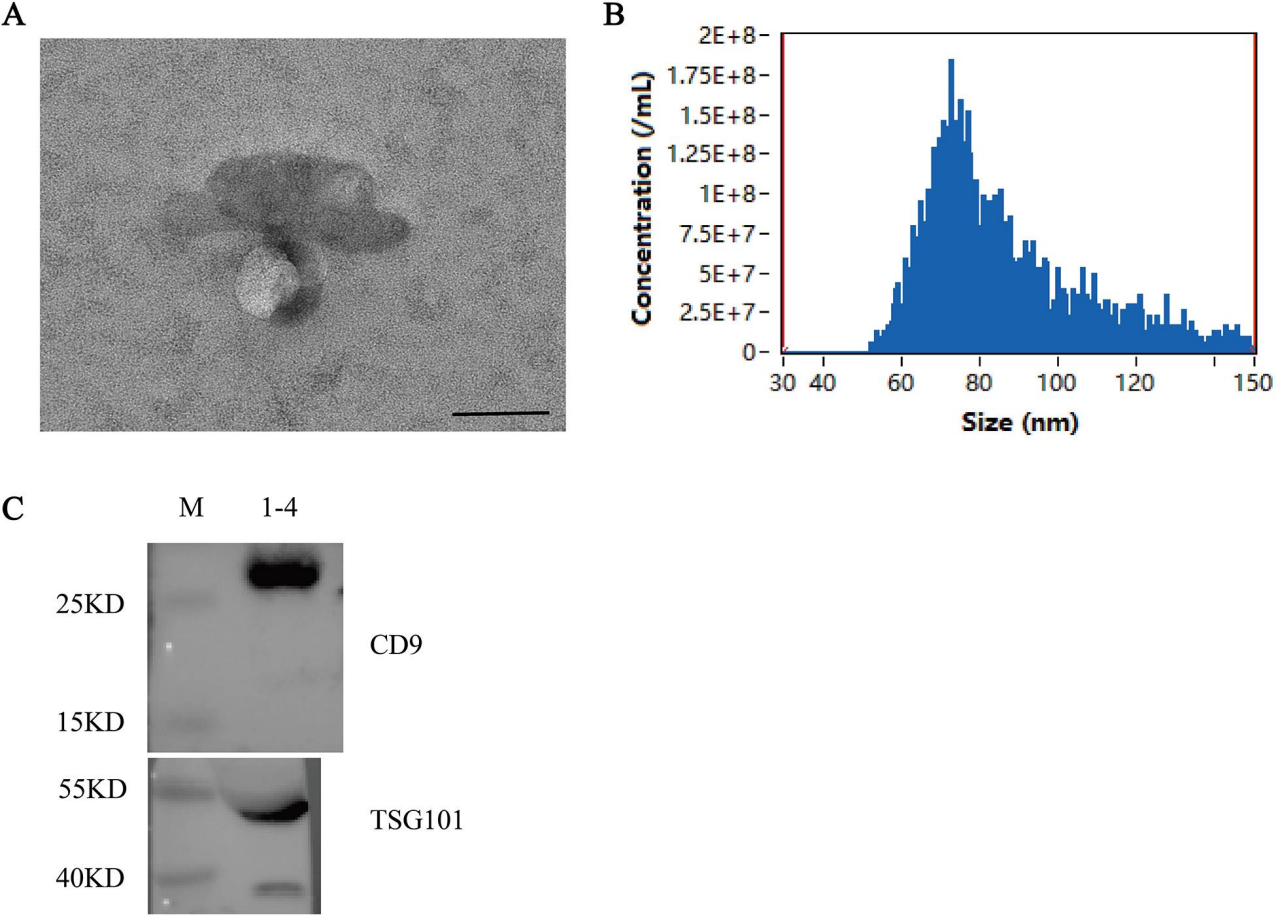
Identification and characterization of exsomes. **(A)** Morphological characterization of isolated serum exosomes by transmission electron microscopy (TEM). The image shows small vesicles of approximately 30–150 nm in diameter, consistent with the typical morphology of exosomes. Scale bar: 100 nm. **(B)** Nanoparticle concentration and size distribution of isolated exosomes. Nanoparticle tracking analysis (NTA, NanoFCM N30E) revealed a peak particle size of 86 nm and a concentration of 8.59 × 10⁹ particles/mL. The histogram illustrates the size distribution profile, confirming that the majority of isolated vesicles fall within the expected exosomal size range (30–150 nm). **(C)** Western blot analysis of exosomal protein markers. Proteins extracted from isolated exosomes were probed for the canonical exosomal markers CD9 (≈25 kDa) and TSG101 (≈55 kDa). Lane M: molecular weight marker; Lane 1-4: exosome sample (protein loading: 100 μg per lane); Lane Positive control: HepG2 cell lysate. Both markers were clearly detected in the exosome fraction, confirming the successful enrichment of exosomes.

Serum exosomes were successfully isolated via differential ultracentrifugation. Transmission electron microscopy (TEM) analysis revealed the presence of cup-shaped membrane vesicles with diameters ranging between 30–150 nm, confirming the typical morphology of exosomes (Figure 2A). Nanoparticle tracking analysis (NanoFCM) further characterized the isolated particles, showing a mean particle size of 86 nm and a concentration of 8.59 × 10⁹ particles/mL (Figure 2B). The size distribution was consistent with the expected range for exosomal vesicles. Protein analysis indicated that the exosome preparation had a protein concentration of 5.32 μg/μL. Western blot analysis confirmed the enrichment of exosomal marker proteins CD9 and TSG101 in the isolated fraction, thereby validating the successful purification of exosomes (Figure 2C).

### Expression profiling of lncRNAs in venous blood from UCM patients pre- and post-renal transplantation

**Figure 3:**
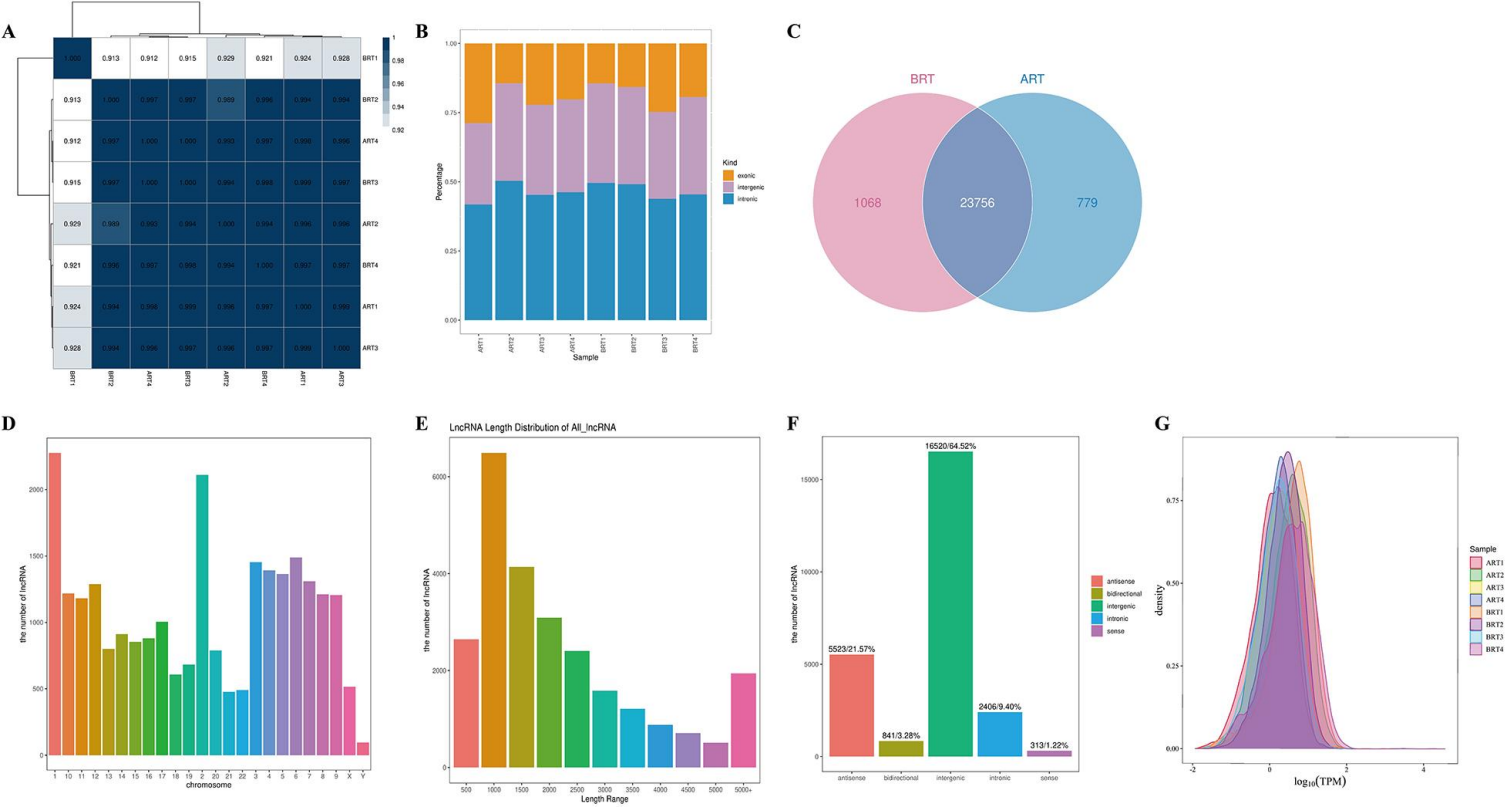
Expression profiles of lncRNAs in the venous blood of UCM patients before and after renal transplantation. **(A)** Heatmap of pairwise sample expression correlations. The x- and y-axes represent sample IDs, ordered according to hierarchical clustering. Dendrograms are displayed at the top and left, and the color scale indicates the degree of correlation between samples, as shown in the legend on the right. **(B)** Histogram showing the distribution of mapped reads across different genomic regions (exonic, intergenic, and intronic) based on the reference genome. Each bar represents a sample. Exonic regions are shown in yellow, intergenic regions in red, and intronic regions in blue. The height of each region indicates the percentage of mapped reads relative to the total mapped reads. **(C)** Venn diagram showing the number of lncRNAs detected in UCM patients before and after renal transplantation. Pink represents pre-transplant, blue represents post-transplant, and purple indicates the overlap. **(D)** Distribution of lncRNA read counts across different chromosomes in each sample. The *x*-axis represents chromosomes, and the *y*-axis indicates the corresponding lncRNA junction read counts. **(E)** Length distribution of lncRNAs. The *x*-axis represents lncRNA length intervals, and the *y*-axis represents the number of lncRNAs within each interval. **(F)** Bar chart showing the proportions of different lncRNA categories. The *x*-axis indicates four types of lncRNAs, and the *y*-axis indicates the number of lncRNAs in each category. **(G)** Comparative TPM density distribution plot for each sample. Different colored curves represent different samples. The *x*-axis indicates the log-transformed TPM values, and the *y*-axis represents the probability density.

To investigate the differences in lncRNA expression before and after renal transplantation, we conducted RNA sequencing on 8 blood samples collected from UCM patients. Correlation analysis could measure the reliability of sequencing results to a certain extent. This provided a key metric for evaluating the reproducibility of biological replicate experiments and the appropriateness of sample selection. Pearson's r > 0.8 denotes ideal experimental and sampling conditions (Figure 3A). We performed statistical analysis on the lncRNAs of each sample and generated distribution histograms of lncRNAs in various regions (exons, intergenic regions, and introns) across different samples (Figure 3B). There were 1068 lncRNAs specifically expressed before renal transplantation, 779 lncRNAs expressed after renal transplantation, and 23756 identical lncRNAs expressed in both groups (Figure 3C). Analysis of all detected lncRNAs revealed that they were most highly expressed on chromosomes 1 (chr1) and chromosomes 2 (chr2), with the lowest expression on the Y sex chromosome (chY) (Figure 3D). Length analysis indicated that almost all lncRNAs were longer than 1000nt, with the majority being intronic (Figure 3E). These consisted of 841 bidirectional lncRNAs (3.28%), 5523 antisense lncRNAs (21.57%), 16,520 intergenic lncRNAs (64.52%), 2,406 intronic lncRNAs (9.40%), and 313 sense lncRNAs (1.22%) (Figure 3F). To assess the overall distribution of lncRNA expression, we measured TPM density distribution. TPM measurements of lncRNAs in both pre-transplant and post-transplant samples ranged from 10-2–102.5 (Figure 2G).

### Differential lncRNA expression profiles

**Figure 4:**
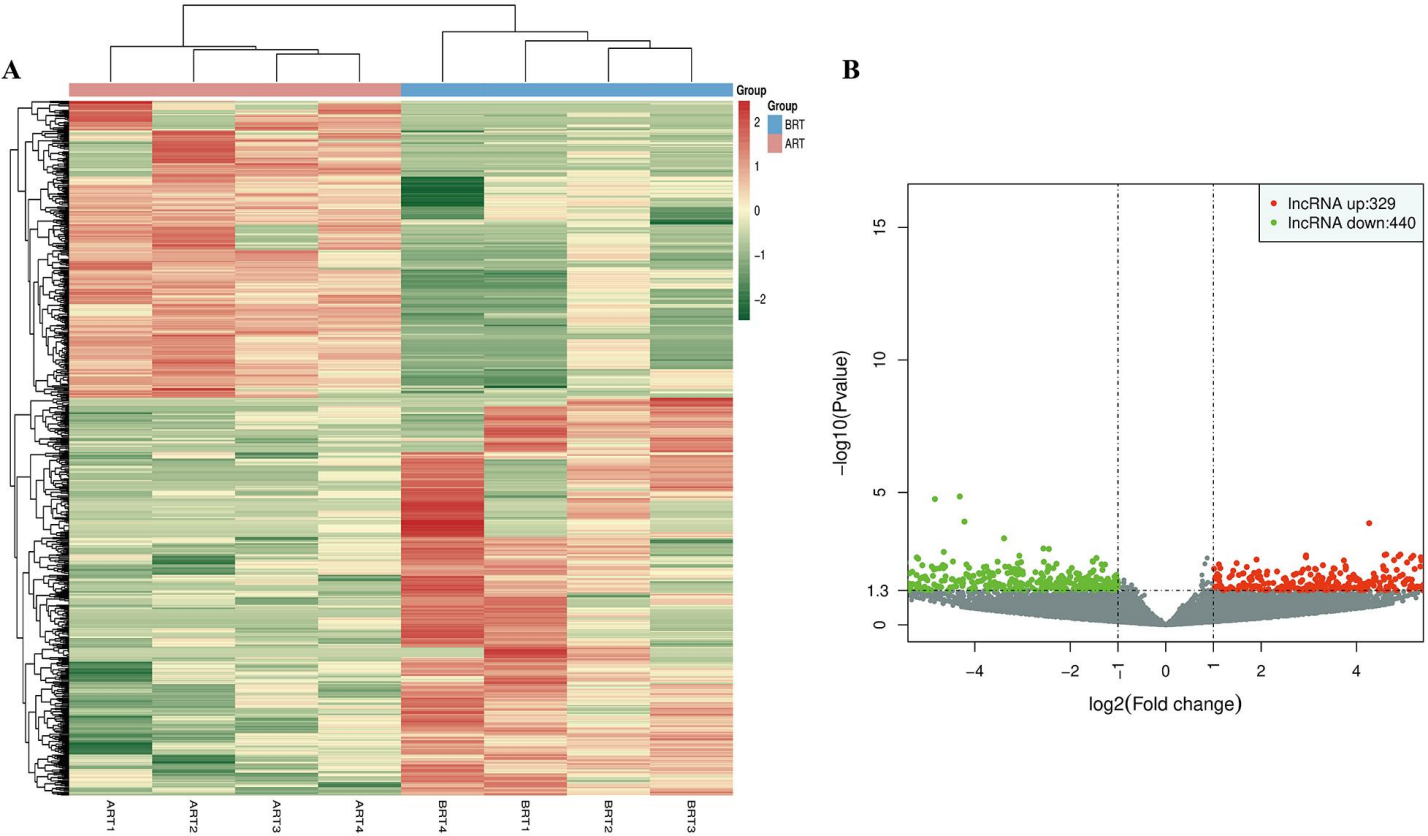
Differential expression of lncRNAs in the venous blood of UCM patients before and after renal transplantation. **(A)** Cluster heatmap of expression patterns of all DElncRNAs (Fold change > 2.0 and *p* < 0.05). The *x*-axis represents sample names and clustering results, while the *y*-axis represents the DElncRNAs and their clustering. Each column corresponds to a sample, and each row corresponds to a specific lncRNA. The color scale indicates the expression level of lncRNAs in the samples, expressed as log2(FC). **(B)** Volcano plot of DElncRNAs (fold change ≥ 2.0, *p* < 0.05). Each point in the volcano plot represents an lncRNA. The *x*-axis shows the log value of the difference of expression levels between the two groups, and the *y*-axis shows the negative log value indicating the statistical significance of the expression change. Larger absolute *x*-axis values represent greater fold changes between groups, and higher *y*-axis values indicate greater statistical significance, suggesting more reliable differential expression. Green dots represent downregulated lncRNAs, red dots represent upregulated lncRNAs, and black dots represent non-differentially expressed lncRNAs.

We analyzed and quantified DElncRNAs, and used heatmaps and volcano plots to illustrate significant differences (Figures 4A, B). Compared to pre-renal transplantation samples, we identified 769 DElncRNAs, including 329 upregulated and 440 downregulated transcripts.

### Prediction of lncRNA target genes

LncRNAs primarily exert their functions by regulating target genes that encode proteins. Therefore, establishing DElncRNAs-mRNAs relationship, coupled with predicting target genes based on the potential mechanisms of lncRNA action, will contribute to further in-depth research on lncRNA functions.

**Figure 5:**
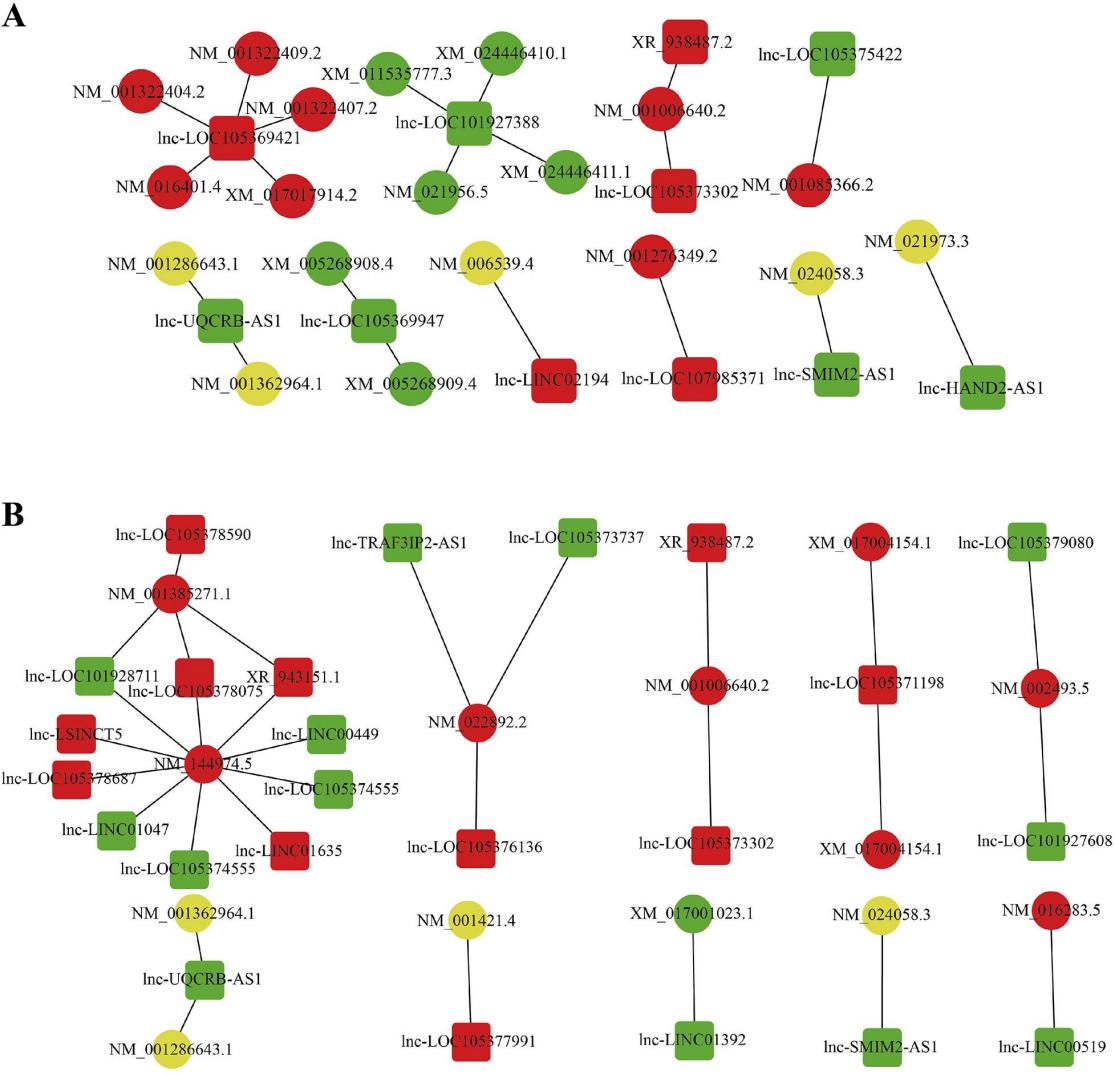
Interaction networks of DElncRNAs and their target genes, as well as lncRNA–mRNA interactions. **(A)** Predicted cis-target genes of differently expressed lncRNAs. **(B)** Predicted trans-target genes of differently expressed lncRNAs. Squares represent lncRNAs and circles represent mRNAs. Red indicates upregulation, green indicates downregulation, and yellow indicates no significant change.

LncRNAs modulate target genes via two primary mechanisms: cis-regulation and trans-regulation. Cis-regulation refers to the process by which lncRNAs exert their biological functions by modulating genes located in close proximity to their own genomic loci. By integrating DElncRNAs with spatially adjacent mRNA data (≤10 kb), we identified candidate cis-acting target genes. Trans-regulation, on the other hand, involves lncRNAs regulating mRNAs located on other chromosomes or at distal regions of the same chromosome. For a subset of lncRNAs, target genes can be predicted based on complementary base-pairing interactions between RNA molecules. To predict trans-regulation, we first extracted the sequences of DElncRNAs and mRNAs, performed an initial screening using the BLAST software, and then applied RNAplex for further filtering, identifying potential target genes of lncRNAs. We selected the 24 significantly up- and down-regulated lncRNAs, and constructed the lncRNA-mRNA regulatory network using the predicted target gene (Figures 5A, B). The results showed that 518 DElncRNAs regulated 529 target genes in cis and 11721 target genes in trans, with one lncRNA regulating up to 142 target genes.

### GO, KEGG pathway and GSEA analyses

**Figure 6:**
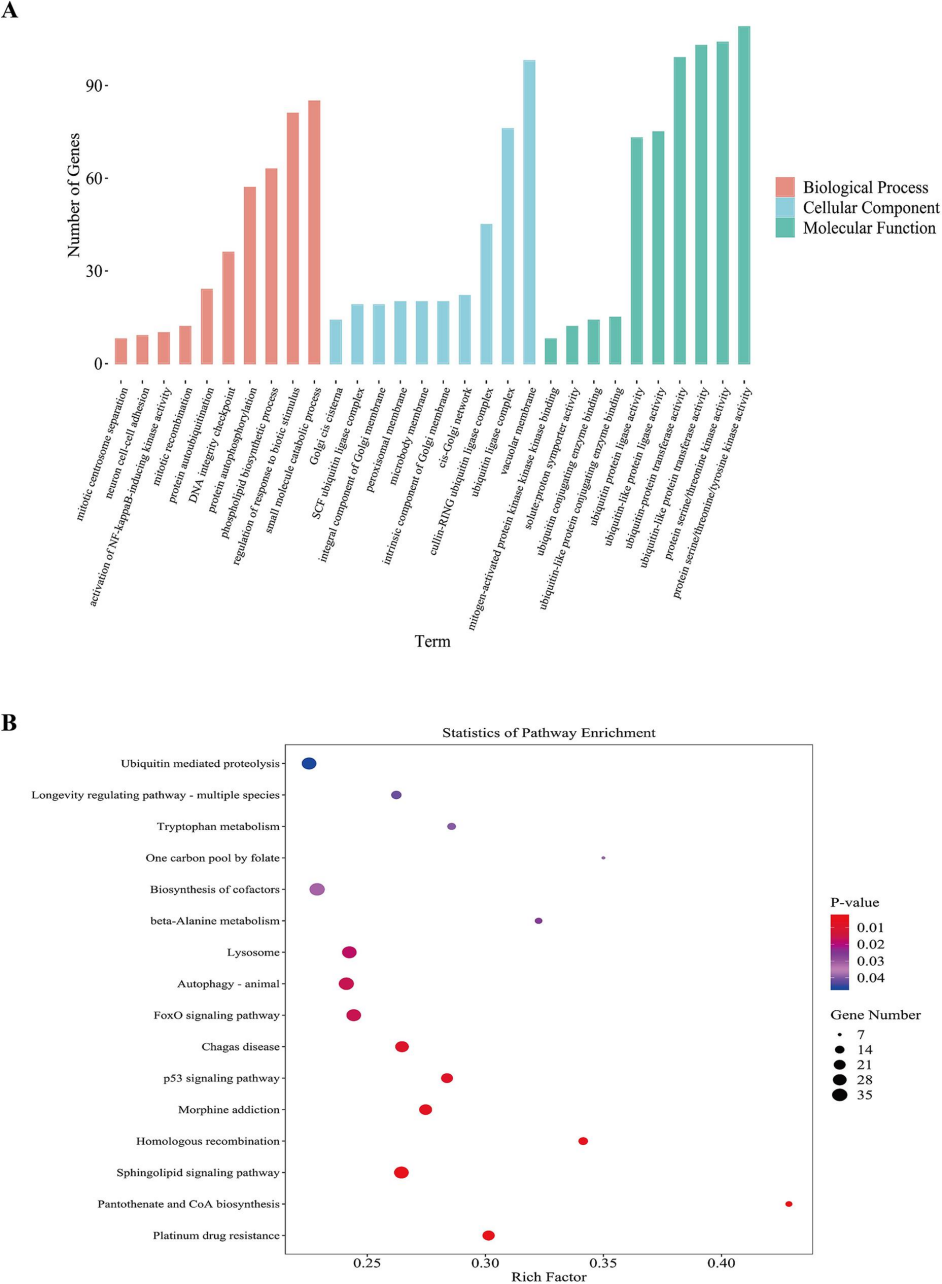
Statistical analysis of lncRNA-derived genes based on GO and KEGG classification before and after renal transplantation. **(A)** Bar chart of GO classification for DElncRNAs before and after renal transplantation. The *x*-axis represents GO categories, and the *y*-axis indicates the number of genes. **(B)** KEGG enrichment bubble plot of lncRNA-derived genes differentially expressed before and after renal transplantation. The *x*-axis shows the GeneRatio, representing the proportion of differentially expressed lncRNA-derived genes annotated in each pathway, and the *y*-axis represents each pathway. The size of each bubble indicates the number of annotated differentially expressed lncRNA-derived genes in the pathway, and the color of the bubble represents the adjusted *p*-value from the hypergeometric test.

**Figure 7:**
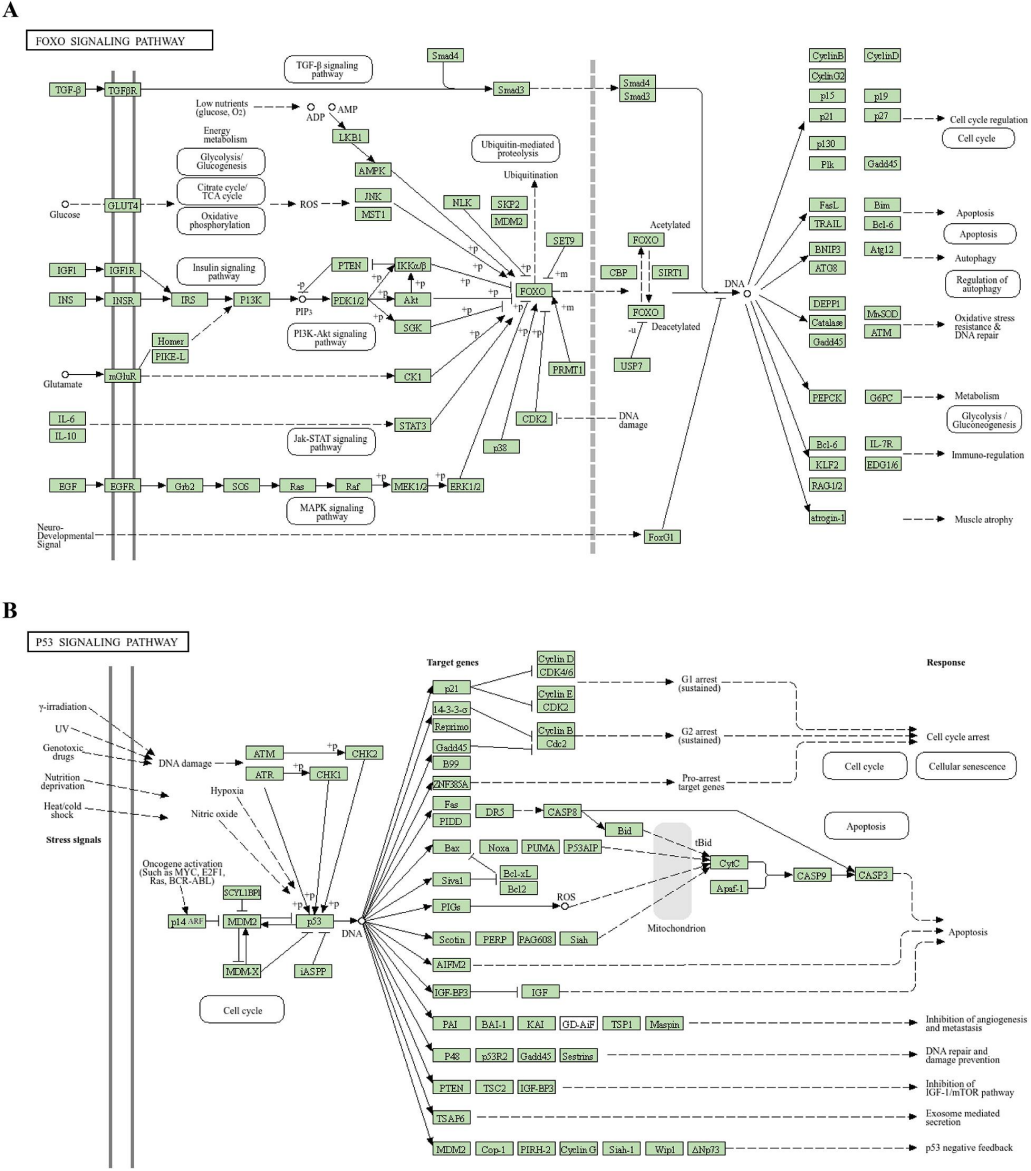
Mapping of the signaling pathway. **(A)** FoxO signaling pathway. **(B)** p53 signaling pathway.

**Figure 8:**
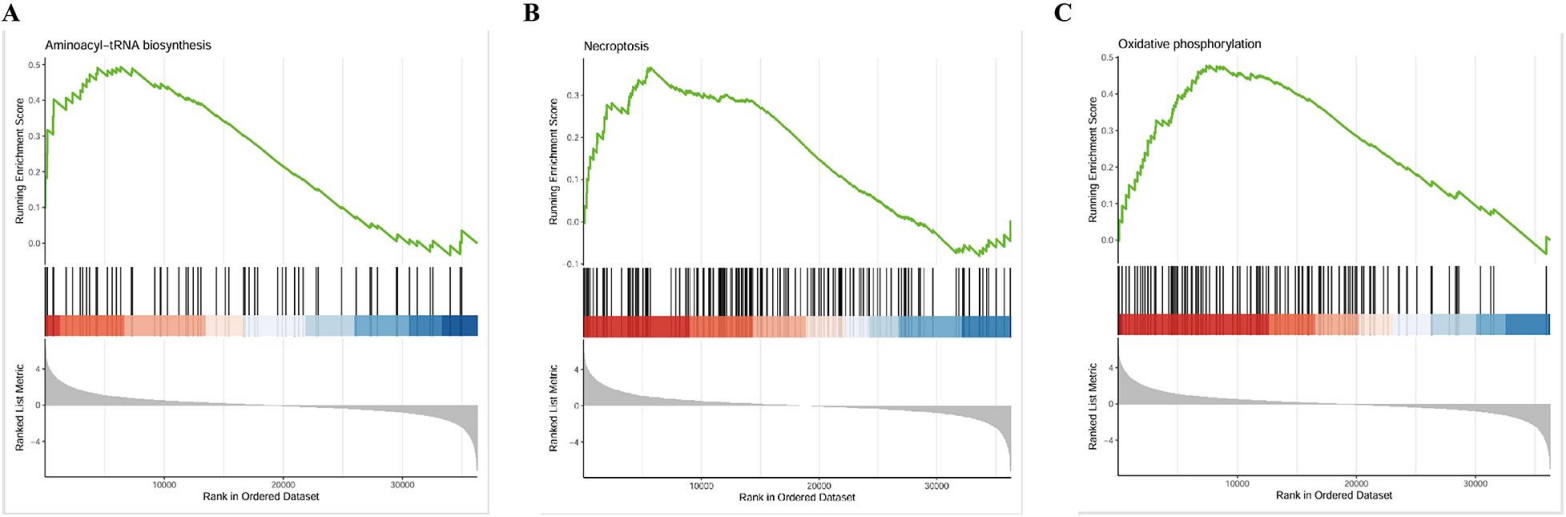
GSEA enrichment analysis results. **(A)** Aminoacyl-tRNA biosynthesis. **(B)** Necroptosis. **(C)** Oxidative phosphorylation.

DElncRNAs (*p* < 0.05) were subjected to GO and KEGG pathway enrichment analyses. For the GO analysis, the top 10 most significantly enriched biological processes, cellular components, and molecular functions were identified (Figure 6A). Among the molecular function categories, the highest GO enrichment was protein serine/threonine/tyrosine kinase activity, which contributed to the pathogenesis of cardiovascular complications associated with renal dysfunction (30–32). Vacuolar membrane exhibited the most remarkable GO enrichment in cellular component. It was revealed that some genes participated in small molecule catabolic process, regulation of response to biotic stimulus, and phospholipid biosynthetic process in biological process category. KEGG pathway analysis revealed significant associations between DElncRNAs and cofactor biosynthesis, sphingolipid metabolism, ubiquitin-mediated proteolysis, FoxO signaling, and p53 signaling pathways (Figure 6B). Activation of the p53 signaling pathway stimulates the fibrosis gene plasminogen activator inhibitor-1, increasing the secretion of fibrosis effectors and accelerating the evolution of renal pathology from acute to chronic phases in experimental mices (33). High levels of indophenol sulfate in the plasma of CKD patients can induce cell aging and death, endothelial cell damage, and promote the development of cardiovascular diseases. Downregulation of the p53 signaling pathway reverses this change, demonstrating a protective effect on cell (34). FoxO signaling activation attenuated renal fibrogenesis and oxidative stress in chronic kidney failure rat models. After upregulating the FoxO signaling pathway in mice with reduced ejection fraction and short axis shortening rate, the decrease in results was mitigated, and the myocardial infarction area was reduced (35). These findings suggested that DElncRNAs might participate in regulating cardiac function before and after renal transplantation through the FoxO and p53 signaling pathway. Figures 7A, B depicted the precise regulatory pathways. Moreover, GSEA showed that aminoacyl-tRNA biosynthesis, necroptosis and oxidative phosphorylation were the most significant enrichment pathways (*p* < 0.01, FDR < 0.05), and Figure 8 presented representative images of the enrichment analysis.

### Validation of identified lncRNAs by RT-qPCR

**Figure 9:**
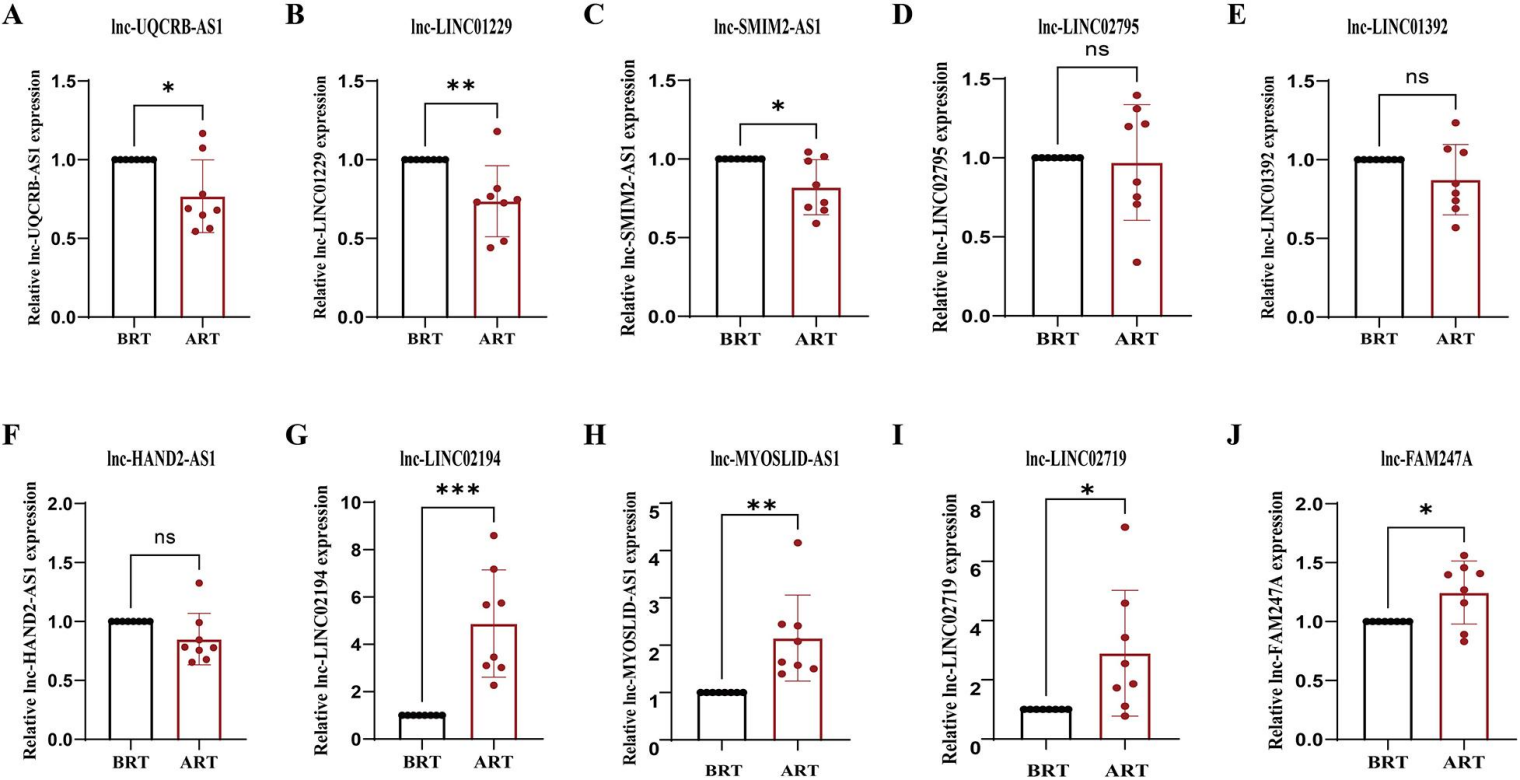
RT-qPCR was used to identify the expression levels of 10 candidate lncRNAs in the before renal transplantation group and the after renal transplantation group. **(A)** lnc-UQCRB-AS1 (*p* = 0.0132), **(B)** lnc-LINC01229 (*p* = 0.0052), **(C)** lnc-SMIM2-AS1 (*p* = 0.0118), **(D)** lnc-LINC02795 (*p* = 0.8240), **(E)** lnc-LINC01392 (*p* = 0.1300), **(F)** lnc-HAND2-AS1 (*p* = 0.0740), **(G)** lnc-LINC02194 (*p* = 0.0003), **(H)** lnc-MYOSLID-AS1 (*p* = 0.0030), **(I)** lnc-LINC02719 (*p* = 0.0240), **(J)** lnc-FAM247A (*p* = 0.0205). The expression levels were quantified and compared between the two groups. Data are presented as mean ± SD. Statistical significance was determined as follows: *p < 0.05, **p < 0.01, ***p < 0.001, ns: not significant. Each experiment was performed twice.

A total of 10 highly DElncRNAs were selected (Tables 1, 2), and their expression levels were quantified via RT-qPCR to validate our sequencing data. Sequencing outcomes indicated that lnc-LINC02194 (*p* = 0.0003), lnc-MYOSLID-AS1 (*p* = 0.0030), lnc-LINC02719 (*p* = 0.0240) and lnc-FAM247A (*p* = 0.0205) were up-regulated after renal transplantation. Lnc-LINC01229 (*p* = 0.0052), lnc-UQCRB-AS1 (*p* = 0.0132), lnc-SMIM2-AS1 (*p* = 0.0118) were downregulated after renal transplantation (*p* < 0.05). Lnc-LINC02194 exhibited the greatest expression difference after renal transplantation (Figures 8A–J). Therefore, the results of RT-qPCR and RNA sequencing are very consistent, indicating the reliability of the expression profile of lncRNA obtained from the sequencing results. Lnc-LINC02194 showed the most significant difference among the validated lncRNAs (Figure 9, Table 3).

**Table 1:** 10 high different-expressed lncRNAs after renal transplantation.

Transcript-position	Gene Name	Regulated	log2FC	*p*-value
chr8:96235782-96239427	UQCRB-AS1	down	−6.287800857	0.002478736
chr16:79795249-79799090	LINC01229	down	−5.692102458	0.003290505
chr1:234678530-234688832	SMIM2-AS1	down	−6.207225141	0.005124291
chr1:86279096-86315502	LINC02795	down	−5.755774888	0.011442853
chr7:115123599-115231355	LINC01392	down	−5.794430378	0.013018536
chr4:173530458-173541932	HAND2-AS1	down	−5.659154289	0.016339857
chr16:24236104-24252860	LINC02194	up	6.193051635	0.001884798
chr2:207186690-207254316	MYOSLID-AS1	up	5.955800663	0.003862689
chr11:106112459-106132116	LINC02719	up	5.889638963	0.004059066
chr22:21192790-21204020	FAM247A	up	5.899569799	0.011243292

**Table 2:** Primers used in present study (5′-3′).

Primer name	Primer sequences
GAPDH-F	GAACGGGAAGCTCACTGG
GAPDH-R	GCCTGCTTCACCACCTTCT
lnc-UQCRB-AS1-F	GGTGATCATAAGGCAGACAACTGA
lnc-UQCRB-AS1-R	CATGTGTTATCCTGGGCAAGTGA
lnc-LINC01229-F	AGCTTGCTATGGGAACACTGGA
lnc-LINC01229-R	CCAGCAGCACCTACTTGTTTGA
lnc-SMIM2-AS1-F	TCCATCCTGGCTCATCTCCTCT
lnc-SMIM2-AS1-R	TCTCACAAAGGCAGTCTGGAGG
lnc-LINC02795-F	AGCGATCCCGCAATTCATTCAT
lnc-LINC02795-R	TCCAGAGGGACCAAGGAACAAG
lnc-LINC01392-F	TGGCAACAGTGACAAATCCTGTG
lnc-LINC01392-R	CAGTGTGCTTGTCACATAGTAGG
lnc-HAND2-AS1-F	TGGCCAAGTGCCTTTCAAACTG
lnc-HAND2-AS1-R	TCACAGCAGCTAATATTGTCCCA
lnc-LINC02194-F	GGGTGCCTTTACCTCTGAAGACT
lnc-LINC02194-R	CCACAGGGCAGAGGTTCAATCA
lnc-MYOSLID-AS1-F	ATACCAGCCATGTGGCATGTCG
lnc-MYOSLID-AS1-R	CTGGTGTTGGTCCTTAGTGTGC
lnc-LINC02719-F	TTCAGGAGCAAAGGCAAGACCT
lnc-LINC02719-R	AATAGCCTTTAGAGCCAGCTTCC
lnc-FAM247A-F	TGCTGCTGACTCCAAGGTCTTC
lnc-FAM247A-R	GGGCATTTCCATTAGCACTCCA

**Table 3:** RT-qPCR results.

Gene Name	Mean of Pre	Mean of Post	Mean (post-pre) ± SD	95% CI	Adjusted P
UQCRB-AS1	1.000	0.7683	−0.2317 ± 0.08165	−0.4068∼-0.05653	0.0132
LINC01229	1.000	0.7363	-0.2637 ± 0.07972	-0.4347∼-0.09276	0.0052
SMIM2-AS1	1.000	0.8206	-0.1794 ± 0.06203	-0.3125∼-0.04640	0.0118
LINC02795	1.000	0.9707	-0.02933 ± 0.1294	-0.3069∼0.2482	0.8240
LINC01392	1.000	0.8727	-0.1273 ± 0.07916	-0.2971∼0.04244	0.1300
HAND2-AS1	1.000	0.8511	-0.1489 ± 0.07715	-0.3144∼0.01652	0.0740
LINC02194	1.000	4.880	3.880 ± 0.8003	2.163∼5.596	0.0003
MYOSLID-AS1	1.000	2.152	1.152 ± 0.3215	0.4624∼1.841	0.0030
LINC02719	1.000	2.901	1.901 ± 0.7508	0.2904∼3.511	0.0240
FAM247A	1.000	1.247	0.2466 ± 0.09446	0.04404∼0.4492	0.0205

To further investigate the correlation between these 10 long non-coding RNAs and echocardiographic cardiac function indicators, we conducted a ROC curve analysis on the PCR-validated differential values of these 10 long non-coding RNAs before and after kidney transplantation, as well as the improvement of cardiac function after kidney transplantation.

The results showed that the area under the receiver operating characteristic curve for lnc-LINC02194 was 0.90.

## Discussion

Exosomes are membrane vesicles approximately 40–100 nm in size, secreted by various cell types into the extracellular space. In recent years, the role of exosomes in the occurrence, development, and treatment of diseases has attracted a lot of attention. The formation of exosomes is a highly regulated process that includes endocytosis, luminal vesicle formation, transport and release. Exosomes represent subproteins and are considered to be non-classical mechanisms of protein secretion. They have been shown to play a role in regulating immune responses, antigen presentation, RNA and protein transfer, and cell-cell (organ-organ) interactions/signaling. Studies have shown that exosomes derived from cardiac progenitor cells can stimulate paracrine molecules involved in cardiac repair mechanisms, including stimulating angiogenesis and inhibiting cardiomyocyte apoptosis. Other studies have shown that exosomes derived from mesenchymal cells can alleviate renal fibrosis and vascular sparsity caused by renal ischemia-reperfusion injury.

LncRNAs represent a class of noncoding RNA molecules with a sequence length greater than 200nt, which participates in disease development by regulating transcriptional silencing, transcriptional activation, and various signaling pathways (36–39). Increasing evidence indicates that DElncRNAs are implicated in the onset of various cardiovascular diseases. Qian Wang et al. identified differentially expressed lncRNAs in the spinal cord of normal and ischemia-reperfusion (I/R) injured rats through RNA sequencing analysis, confirming that key lncRNAs (such as upregulation of NONRATT025386 and downregulation of NONRATT002188) may be a new target for treating I/R-induced cardiac injury (40). Huaping Li et al. conducted microarray analysis on 14 cases of Dilated cardiomyopathy (DCM) and 10 control human heart samples, revealing significant differential expression of lncRNAs RP11-54D21.2 and XLOC_014288, which may be associated with left ventricular function in DCM (41). However, limited studies have explored the role of lncRNAs in UCM. In this study, venous blood specimens from UCM patients were collected both pre- and post-transplantation for gene sequencing. Echocardiography showed that, after renal transplantation, cardiac hypertrophy in UCM patients reduced, and echocardiographic indicators of ventricular contraction and relaxation functions improved. Using next-generation sequencing, a total of 23,756 lncRNAs and 769 DElncRNAs were identified. Therefore, it is plausible that these DElncRNAs may play a role in the progression of UCM.

It is worth noting that while our study utilized a relatively small cohort size (*n* = 4 pairs), we adopted a rigorous longitudinal paired design to maximize statistical power and biological relevance. By treating each patient as their own control, this design effectively eliminates inter-individual variability caused by genetic background, age, and baseline comorbidities, which are major confounding factors in heterogeneous patient populations. Furthermore, to mitigate the risk of false positives associated with the limited sample size, we implemented a strict multiple testing correction using the Benjamini–Hochberg (BH) procedure. This approach ensures that the identified differentially expressed lncRNAs represent robust molecular signatures specifically linked to the therapeutic intervention rather than random biological noise.

GO enrichment analysis revealed significant associations between differentially expressed genes and three key biological processes: biotic stress response regulation, ubiquitin ligase complex assembly, and serine/threonine/tyrosine-specific protein kinase activity. KEGG analysis indicated that more than 10 pathways were associated with UCM. Down-regulation of p53 signaling pathway can prevent cell death, aging, damage to angiogenic activity, and even reverse the progression of cardiovascular diseases (34). Loss of the p53 signaling pathway can reduce the secretion of renal tubular fibrosis effectors in mice, thus slowing the progression from acute to chronic kidney injury (33). Activation of the FoxO signaling axis attenuates renal interstitial fibrogenesis and oxidative damage in CKD and alleviates ischemia-reperfusion injury in myocardial cells (35, 42). Fibrotic remodeling commonly results from endothelial dysfunction induced by inflammatory and oxidative damage, and plays a major role in the mechanisms underlying cardiorenal syndrome (43). Therefore, we infer that the p53 and FoxO signaling pathways may be critical for improving cardiac function in UCM patients after renal transplantation and could function as crucial therapeutic targets for reducing cardiovascular events in these patients.

LncRNAs can exert powerful biological effects through direct regulation of mRNA. To dissect the underlying regulatory landscape of UCM, we constructed a lncRNA-mRNA targeted network featuring cis and trans regulation. The evaluation of the lncRNA-mRNA interaction network will facilitate deeper investigation into the predicted lncRNA downstream targets and their potential mechanism of action. This research found that lnc-LOC105379080 and lnc-LOC101927608 upregulated NDUFB6(NM_002493.5) via trans-regulation. NDUFB6 encodes a protein belonging to mitochondrial complex I, which is the core subunit of respiratory chain complex I (RCI). The respiratory chain is the main source of reactive oxygen species and plays a crucial role in regulating oxidative stress. When respiratory chain function is impaired, tissues with high energy metabolism demands—such as the heart—are most vulnerable. This dysfunction can lead to pathological manifestations including left ventricular hypertrophy, heart failure, and ischemia-reperfusion injury (44). Dysfunction of mitochondrial respiratory chain complex can lead to mitochondrial fusion, structural damage to renal tubules, glomeruli, and renal papilla, inhibition of renal cell proliferation, and thus renal dysfunction (45). Following renal transplantation, upregulation of NDUFB6 improves myocardial oxygen supply, which is beneficial for reducing myocardial hypertrophy and improving heart function. GSEA shows that oxidative phosphorylation, the final step of the respiratory chain, is one of the most significant enrichment pathways, confirming that NDUFB6 affects the structure and function of both the heart and kidney through energy metabolism, and is a key gene in heart kidney syndrome. In addition, downregulated lnc-LOC105369947 reduces NFYB(XM_005268909.4, XM_005268908.4) gene expression through cis regulation. The absence of NFYB-1 leads to mitochondrial gene expression disorders, reduced oxygen consumption, mitochondrial breakage, and disruption of mitochondrial stress pathways (46). These changes will exacerbate renal fibrosis by promoting epithelial mesenchymal transition (47). Based on these studies, we speculate that lnc-LOC105379080, lnc-LOC101927608, and lnc-LOC105369947 may be associated with specific mRNA targets and potentially correlate with UCM progression.

RT-qPCR validation confirmed that lnc-LINC02194 was significantly upregulated after renal transplantation, consistent with RNA sequencing results. Thus, we hypothesized that lnc-LINC02194 is one of most important lncRNAs associated with the onset and progression of UCM. According to lncRNA-mRNA interaction analysis, CACNG3(NM_006539.4) may represent a potential downstream target gene. CACNG3 is implicated in the progression and prognosis of multiple diseases while suppressing gliomagenesis and progression through modulation of synaptic transmission and specific neurotransmitter signaling pathways (48). A recent study has identified abnormal expression of CACNG3 in breast cancer (49). Another study found that CACNG3 exhibited a higher coefficient in advanced stages of pancreatic cancer (50). Notably, CACNG3's contribution to cardiopathology and renal physiology remains underexplored. Future studies should focus on elucidating the function of specific lncRNAs and their regulatory mechanisms in UCM.

This investigation is constrained by a limited clinical cohort, necessitating expanded validation in larger populations to corroborate the findings. Secondly, the study solely verified DElncRNAs in clinical specimens but lacks experimental validation in UCM cell lines and animal models to elucidate the functional mechanisms of these regulatory molecules. Consequently, Future research involving *in vitro* knockout/overexpression experiments or exosome transfer assays is necessary to confirm the functional roles of these lncRNAs.

## Conclusion

In summary, an aggregate of 769 DElncRNAs were identified in venous blood specimens from UCM patients both pre- and post-renal transplantation. These lncRNAs may be associated with the regulation of cardiac function by the kidneys and the progression of UCM. This research reveals novel aspects of the potential pathogenesis of UCM before and after kidney transplantation. Future researches may further clarify the functional roles and mechanistic contributions of lncRNAs to UCM pathogenesis.

## Data Availability

The datasets presented in this study can be found in online repositories. The names of the repository/repositories and accession number(s) can be found in the article/Supplementary Material.
