# Supplementary material for: Comprehensive lnc-RNAs expression profiles in uremic cardiomyopathy before and after renal transplantation
Source: Front Cardiovasc Med. 2026 Mar 12;13:1718529. doi: 10.3389/fcvm.2026.1718529 (PMC13017380; doi:10.3389/fcvm.2026.1718529)
Supplement: Supplementary file 1 [file Table1.docx]

**Table S1: Clinical information of patients in sequencing and RT qPCR groups**

| Patient ID | Age(Y) | Sex | Primary disease | Dialysis vintage(M) | Immunosuppressants | eGFR-pre | eGFR-post | Time interval(D) | Post-transplant time(D) |
| --- | --- | --- | --- | --- | --- | --- | --- | --- | --- |
| P1 | 45 | Female | Polycystic kidney | 12 | Tacrolimus | 15 | 99 | 125 | 123 |
| P2 | 53 | Male | Hypertension | 7 | Tacrolimus | 19 | 100 | 126 | 125 |
| P3 | 49 | Male | Type 2 diabetes mellitus | 16 | Tacrolimus | 11 | 104 | 119 | 117 |
| P4 | 55 | Female | Nephrotic syndrome | 9 | Tacrolimus | 18 | 98 | 124 | 122 |
